# Toward a Deeper Understanding of the Seaweed Sensory Space Through Consumer‐Evoked Associations Across Ireland, Norway, Spain, and Turkey

**DOI:** 10.1002/fsn3.72508

**Published:** 2026-10-26

**Authors:** Gonzalo Garrido‐Bañuelos, Guro Møen Tveit, Hanne Dalsvåg, Solveig Uglem, Marta Martínez‐Sanz, Patricia Lopez‐Sanchez

**Affiliations:** ^1^ Innovate Solutions Galway Ireland; ^2^ School for Data Science and Computational Thinking Stellenbosch University Stellenbosch South Africa; ^3^ Department of Fisheries and New Biomarine Industry SINTEF Ocean Trondheim Norway; ^4^ Instituto de Investigación en Ciencias de la Alimentación (CSIC‐UAM) Madrid Spain; ^5^ Department of Food Technology Instituto de Investigaciones Marinas‐Centro Superior de Investigaciones Científicas (IIM‐CSIC) Vigo Spain

**Keywords:** consumers, cross‐cultural study, seaweed, sensory

## Abstract

Understanding the sensory space of a product category is a valuable tool in New Product Development, particularly for emerging food categories such as seaweed, where consumer familiarity remains limited in many markets. The current work explored the sensory associations of consumers willing to try seaweed products across four European countries (Ireland, Norway, Spain, and Turkey) through a cross‐cultural online survey. Participants assessed five organoleptic dimensions (color, visual appearance, flavor, basic tastes, and texture‐mouthfeel) as positive or negative factors and Check‐All‐That‐Apply (CATA) methodology to understand what individual sensory attributes participants associate with seaweed. Besides country of origin, participants' answers were split into perceptuals (those who had previously eaten seaweed products) and conceptuals (those who had not). Results from two‐way ANOVA, Multiple Factor Analysis, and Agglomerative Hierarchical Clustering showed that both country of origin and previous exposure to seaweed products significantly influenced sensory associations across all dimensions. Polarity analysis identified “metallic”, “chewy”, and “sticky” as attributes associated with negative perception, whereas “creamy”, “soft”, and “seafood‐like” showed positive valence across countries. These findings contribute novel and practical insights for product developers targeting the increase in seaweed consumption among European consumers.

## Graphical abstract

A cross‐cultural survey in Ireland, Norway, Spain and Turkey mapped consumers' sensory associations with seaweed. Both country of origin and previous seaweed consumption (perceptual vs. conceptual) shaped these associations, offering practical guidance for developing seaweed products for European consumers.

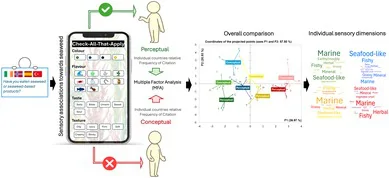

## 1 Introduction

In New Product Development (NPD), a vague sensory space of a product category can create a gap between the industry and end users (i.e., consumers), leading to higher risk of market failure. Understanding the sensory space of a product category can help Food Industry to identify the barriers and drivers of liking of these products from different targeted group of consumers (Collier et al. 2024; Costa et al. 2023; Saldaña et al. 2019). To date, the number of peer reviewed sensory studies applied to seaweed is limited to 91 (17 being analytical and 74 of hedonic or mixed nature), with a third of these works having been published since 2021 (Dahlstedt et al. 2025).

In the context of seaweed, a broad and diverse product category with growing commercial interest, a well‐defined sensory space is particularly valuable and to date, despite the increasing interest within the global Food Industry and Culinary Sciences (Astorga‐España and Mansilla 2013; Figueroa, Farfán, and Aguilera 2023; Mouritsen et al. 2018; Mouritsen et al. 2012; Mouritsen, Rhatigan, and Pérez‐Lloréns 2019), the sensory space of these raw materials remains relatively unexplored. Consumer studies have highlighted flavor as a key parameter to promote seaweed consumption. In a study by Wendin and Undeland (2020) (Wendin and Undeland 2020) exploring the attitudes and preferences of seaweed as food products among Swedish consumers, 60% of participants would consider eating seaweed because they are “good for the environment”, “healthier” and “tasty”. On the other hand, taste seems to be a double driver, since it was also found to be a driver (“not tasty”) for consumers who would not consider eating seaweed products (Wendin and Undeland 2020). Similarly, Jönsson et al. (2026) found that despite over 95% of Swedish participants expressing interest in seaweed, consistent with results from Wendin and Undeland (2020), only 41.2% consumed it regularly (monthly or more often). This gap can potentially be driven by health concerns, sustainability aspects and sensory perceptions. For the latter, related to this current work, participants associated seaweed with saltiness, fresh sea and umami, with most participants showing preference of incorporating seaweed in savory dishes or to be used as flavor enhancers.

Seaweed species have a wide compositional and structural variability (Ferreiro et al. 2026), however, they are broadly classified into brown, green, and red seaweed according to their pigment composition. Yet, the color hue is diverse and complex among different seaweed species. As an example, within red seaweed, *Pyropia and Porphyra* are described as red whereas *Palmaria palmata* is described as magenta (Dahlstedt et al. 2025; Jönsson et al. 2023).

There are thousands of different seaweed species; only in Europe, there is evidence of consumption of over 100 edible species according to Mendes et al. (2022), and this number becomes closer to 650 edible seaweeds at a worldwide level, mainly in Asian countries. Having this diversity into consideration, it is more than likely that there would be a wide sensory space in food products containing seaweed among their ingredients.

Seaweed smell and taste/flavors may be both positive and negative. Dahlstedt et al. (Dahlstedt et al. 2025) showed that certain organoleptic properties were shared among the different seaweed groups (i.e., brown, green and red seaweed) and, that the presence of specific organoleptic properties was associated to individual species for example, the smell of “fungi” was only found to be associated to *Ulva rigida* , Another singular sensory feature was the smell of “shellfish”, only associated to *Palmaria palmata* . Conversely, terms such as “caramel”, “grass” or “herbal”, among others, have been reported in several species. The term “caramel” is a smell and flavor characteristic associated to *Durvillacea antartica* (brown seaweed) and *Pyropia* and *Porphyra* spp. (red seaweed) (Figueroa, Bunger, et al. 2022). In the case of the terms “grass” and “herbal”, the former was a smell and flavor associated to *Alaria esculenta* (brown) and *Ulva* sps. (green), whereas the latter (“herbal”) was found associated to *Pyropia* and *Porphyra* (red), and in *Ulva lactuca* (green). Similarly, a report on Irish seaweed species described Egg wrack ( *Ascophyllum nodosum* ) as unpleasant, with a strong and distinctive marine flavor (Hotchkiss 2007). Processing did not improve Egg wrack flavor, as it was perceived as negative in dry and wet forms. In the same report, other species such as *Himanthalia elongata, Saccharina latissima* and *Ulva rigida* also had a negative flavor perception in their dry form.

Seaweeds follow a more similar pattern regarding the basic tastes: high saltiness, medium umami, low bitterness and sourness, and no sweetness as described in the study by Jönsson et al. (2023). The fifth basic taste, umami, is a core matter of research to understand seaweed flavor potential (Figueroa, Bunger, et al. 2022; Milinovic et al. 2020, 2021; Mouritsen, Duelund, et al. 2019). Linked to the amount of monosodium glutamate (MSG) in seaweed (Figueroa, Bunger, et al. 2022), the umami perception can be enhanced with the presence of some volatile compounds (Frøst et al. 2021). The levels of glutamic acid or aspartic acid may also contribute, but a lower correlation was found in the study by Jönsson et al. (2023). The level of saltiness is linked to their level of sodium, and Jönsson et al. (2023) showed that seaweed in powder form were perceived as saltier than as a whole. This is why seaweeds are considered a potentially good alternative for salt replacers. The Irish report by Hotchkiss (2007) indicated that Atlantic wakame ( *Alaria esculenta* ), Dulse ( *Palmaria palmata* ), Irish nori (*Porphyra*), and Sea lettuce ( *Ulva rigida* ) have potential to be used as salt replacers (Hotchkiss 2007). In terms of texture, seaweed sensory spectrum also covers a wide range of terms including “chewy”, “firm”, “crispy”, “viscous”, “astringent” or “sticky”. The term “chewy” was associated to green ( *Ulva rigida* ) and red seaweed species (*Pyropia, Porphyra and Palmaria palmata*) (Dahlstedt et al. 2025).

All these sensory properties are linked to changes in the chemical and structural composition, such as polysaccharide and protein content (Souto‐Prieto et al. 2024), and inter‐species variability which leads to a potentially wide sensory spectrum. Additionally, the type of processing (Figueroa, Holdt, et al. 2023; Garrido‐Bañuelos et al. 2022; Stévant et al. 2018), cooking techniques (Bingi et al. 2025; Sánchez‐García et al. 2020), and storage (Stévant et al. 2020) can also influence seaweed sensory properties. Understanding the seaweed sensory potential is the first step, to be followed by understanding its interaction with other ingredients in different food matrices. To date, many studies have investigated the addition of seaweed as functional ingredients added to food products such as bread and spreads (Jönsson et al. 2024), vegan burgers (Fernandes et al. 2023), or pork liver pâté (Agregán et al. 2018).

The current work aims to provide further insights to the current knowledge about seaweed sensory space. These insights are not extracted from a sensory evaluation of specific seaweed or seaweed‐based products but from a cross‐country online survey. Our main research hypothesis is based on the idea that exposure may influence the sensory associations, either understood as individual consumer past experiences or as cultural (i.e., country) exposure to seaweed products. Thus, the goal is to explore what the current sensory associations consumers have toward seaweed in four European countries (Ireland, Norway, Spain and Turkey), and whether these associations are influenced by the country of origin, individual past exposure or both.

## 2 Material and Methods

This work is part of a three‐section online survey that was conducted in Ireland, Norway, Spain and Turkey (Garrido‐Bañuelos et al. 2026). Participants were part of Innovate Solutions private consumer database in Ireland, and TGM Research was used as a third party to access their national databases in the local markets of Norway, Spain and Turkey. Prior to the start of the project, ethical suitability was evaluated via a self‐assessment conducted as part of the project proposal submission. Project approval was subsequently granted under the EU Sustainable Blue Economy Partnership (Horizon Europe Programme), contingent on this assessment. Data were collected through an anonymous online survey, and no personally identifiable information was obtained from participants. Participation was voluntary, and completion of the survey was taken as implicit informed consent.

Data was captured using SurveyMonkey cloud‐based software (SurveyMonkey Inc., San Mateo, California, USA). The original questionnaire was set up in English, with official translation to local languages by TGM. Results from all four countries were centralized. The initial survey captured a balanced proportion between genders, and a similar balance between all countries, with the largest percentage of participants falling into middle age groups (25–34, 35–44, and 45–54 years old) (Garrido‐Bañuelos et al. 2026). Due to the overall goal of the project, at the end of the first section, the following disqualifying question was included: “Are you willing to try seaweed products?” Participants not willing to try seaweed were excluded. The current work focuses on that subset of participants who were willing to try seaweed products in all four countries: Ireland (*n* = 711), Norway (*n* = 763), Spain (*n* = 1075), and Turkey (*n* = 937). Gender and distribution remained balanced after the disqualification question (Table S1).

### 2.1 Consumer Segmentation by Previous Seaweed Exposure

Following the rationale used by Ballester et al. (Ballester et al. 2008) to explore wine sensory spaces, participants were split into “perceptuals” and “conceptuals”. This split was a result of the following question: “have you eaten seaweed or seaweed‐based food products?” In our case, perceptuals were those participants who had eaten seaweed products and whose associations were built upon a real sensory experience, whereas conceptuals were those participants who had never eaten seaweed products, and whose associations are based on preconceived concepts. For perceptuals, frequency of consumption was asked (“how many times?”) using a single select format with four options: “only once”, “few times”, “several times”, and “I am a regular consumer”.

#### 2.1.1 Positive & Negative Perceptions of Seaweed Sensory Dimensions

Potential sensory drivers and barriers were explored evaluating five sensory dimensions “as a positive factor” or “as a negative factor” (Q1). The question was slightly rephrased to perceptuals (“*how do you perceive…?”*) and conceptuals (“*what is your idea about…?”*) followed by color, visual appearance, flavors, taste, and mouthfeel (Table 1).

**TABLE 1:** Survey questionnaire capturing positive and negative perception of five organoleptic dimensions and associations of individual sensory attributes using Check‐All‐That‐Apply (CATA).

How do you perceive/What is your idea about seaweed…?		
QQ1		
Color		As a positive factor
Visual appearance		As a negative factor
Flavor		
Taste		
Texture‐mouthfeel		
Individual sensory attributes		
QQ2		
Blue	Black	Maximum 1 choice.
Green	White	
Brown	Magenta	
Red	Orange	
Golden/Yellow		
QQ3		
Marine	Honey	Restricted CATA (Up to 5 attributes).
Herbal	Truffle	
Earthy/moldy	Smoky/Roasted	
Mineral	Vegetable smell	
Caramel	Fruity	
Fishy	Metallic	
Tea‐like	Rotten	
Meaty	Rancid	
Seafood‐like	Spicy	
	Grassy	
QQ4		
Bitter	Sweet	CATA
Sour	Umami	
Salty		
QQ5		
Oily	Chewy	CATA
Juicy	Creamy	
Firm	Particulated	
Soft	Sticky	
Astringent	Viscous	

#### 2.1.2 Seaweed Individual Sensory Associations

The individual sensory attributes evaluated using pick‐K‐attributes and Check‐All‐That‐Apply (CATA) questionnaire (Adams et al. 2007) are displayed on Table 1. The number of terms can have an impact on consumers' assessment with 20 to 28 terms being considered a “long” sensory ballot (Jaeger et al. 2015). Therefore, to avoid participants' overwhelm, the CATA questionnaire was split according to color, flavor, taste, and mouthfeel properties.

The number of possible answers to color and flavor were restricted. For color, a total of nine different colors (Table 1) were assessed according to existing literature (Figueroa, Bunger, et al. 2022; Jönsson et al. 2023), and participants were asked to select only one option according to the question: “what is the first color you associate to seaweed?”(Q2). Similarly, flavor associations were captured through a total of 19 predefined sensory attributes (Figueroa, Farfán, and Aguilera 2023; Figueroa, Holdt, et al. 2023; Figueroa, Bunger, et al. 2022; Hotchkiss 2007; Jönsson et al. 2023), with participants asked to select up to five sensory attributes in response to “what are the main flavors that you associate to seaweed? (select up to 5)” (Q3). Selection was limited to five attributes to focus on the main five and potentially most relevant attributes; therefore, reducing the risk of over‐attribute selection which may only represent noise. For basic tastes (Q4) and mouthfeel properties (Q5), participants were asked to select all the parameters that apply (Table 1). Mouthfeel attributes were defined from existing literature in seaweed (Figueroa, Bunger, et al. 2022; Hotchkiss 2007; Jönsson et al. 2023) and texture‐mouthfeel attributes in other food systems (Garrido‐Bañuelos et al. 2023; Mihnea et al. 2022; Vickers et al. 2015).

### 2.2 Data Analysis

#### 2.2.1 Seaweed Sensory Properties According to Country of Residence and Previous Experience

Despite following a non‐normal distribution, Meyners and Hasted (Meyners and Hasted 2021) proved that ANOVA can be considered a suitable approach to analyze CATA data when having a sample size > 100 counts. This approach was recently used in a cross‐country study on sustainable food packaging (Norton et al. 2026). Cross‐country differences on sensory dimension and CATA data were firstly assessed through 2‐way ANOVA, considering participants country of origin (Ireland, Norway, Spain and Turkey) and, experiences with seaweed products (i.e., perceptuals and conceptuals), with pairwise‐comparisons between mean values being done with Tukey's HSD test. For the assessment of the different sensory dimensions, data was converted into a binary tale with 1 representing “positive” and 0 “negative”.

To explore the relationship between countries and past experiences with the proposed sensory variables, the obtained average values were analyzed using Multiple Factor Analysis (MFA) (Abdi et al. 2013). For this test, four individual tables corresponding to the four sensory dimensions evaluated differently (i.e., color—C, flavor—F, basic tastes—T, and texture‐mouthfeel—M) were considered. Based on relevant findings, a more in‐depth and separate comparison was also conducted for flavor sensory attributes with Agglomerative Hierarchical Clustering (AHC) done on the average values, and wordclouds based on individual sensory attribute frequency of citation (FoC) integrated within the subsequent dendrograms. Data was analyzed using XLSTAT (version 2023.3.1.1416, New York, USA).

#### 2.2.2 Polarization of Flavor and Texture‐Mouthfeel Sensory Attributes

For flavor and texture‐mouthfeel, the citation counts of each individual attribute were compared between participants who perceived the corresponding sensory dimension as positive and those who perceived it as negative. To reduce noise, sensory attributes with average rFoC lower than 5% were excluded. Therefore, the polarity of each attribute was calculated following a Bayesian Beta‐Binomial model with a weakly informative Beta (1,1) prior (Gelman et al. 2013) for each individual sensory attribute of flavor and texture‐mouthfeel dimensions. This calculation used the citation, and non‐citation counts from the positive and negative perception groups as input, and 200,000 simulated draws per group to estimate the probability of being positive. This was implemented using the *abtestwise* Python package (version 3.13.15; pandas, version 2.2.3; NumPy, version 2.1.3) and was run in Google Colab.

These results were visualized by plotting the average relative frequency of citation (rFoC) for each individual attribute against the ratio of positive/negative perception citations. The Bayesian probability of each attribute being positive is represented in Figure 4 as a continuous color gradient (viridis color scale), with yellow indicating a high probability of positive association and purple indicating a low probability (i.e., stronger association with negative perception). An attribute was considered polarizing when it combined a high overall citation frequency (rFoC) with a low probability of positive association, indicating that there are a substantial proportion of participants on both sides (positive and negative).

## 3 Results and Discussion

The following sections will describe the organoleptic associations split into two consumer segments: whether participants self‐reported to have previously consumed seaweed or seaweed‐based products or not (“perceptuals” and “conceptuals”). This segmentation focuses on food product exposure rather than familiarity with the raw material (i.e., seaweed) itself. This choice was based on the fact that participants could have a different level of familiarity subjected to other factors not investigated in this survey.

### 3.1 Overall Sensory Perception

Table 2 summarizes how participants perceive the following organoleptic dimensions: color, visual appearance, flavor, taste, and mouthfeel properties in seaweed. For almost all participants and dimensions, a positive perception was obtained, except for conceptual Irish participants in the mouthfeel dimension (43.2% positive vs. 56.8% negative) and visual appearance (45.7% positive vs. 54.3% negative). A positive trend can be immediately appreciated, but it is worth highlighting that all participants had previously stated that they were willing to try seaweed or seaweed‐based products. Results from ANOVA analysis showed how country of origin and previous exposure had a significant difference in all five dimensions.

**TABLE 2:** Percentage distribution of responses across groups (countries and exposure) to show the positive and negative perceptions of five organoleptic dimensions.

		Ireland		Norway		Spain		Turkey	
		Co.	Pe.	Co.	Pe.	Co.	Pe.	Co.	Pe.
Color	**−**	31.5%	15.7%	30.4%	19.7%	23.1%	15.3%	23.8%	12.1%
	+	68.5%	84.3%	69.6%	80.3%	76.9%	84.7%	76.2%	87.9%
*F* = 11.8; *p* < 0.0001		d	b	d	bc	c	ab	c	a
Visual appearance	**−**	54.3%	28.1%	49.0%	36.1%	39.0%	25.8%	27.2%	16.5%
	+	45.7%	71.9%	51.0%	63.9%	61.0%	74.2%	72.8%	83.5%
*F* = 27.1; *p* < 0.0001		c	b	d	c	c	b	b	a
Flavor	**−**	24.7%	12.8%	29.7%	15.3%	27.1%	11.7%	14.9%	8.2%
	+	75.3%	87.2%	70.3%	84.7%	72.9%	88.3%	85.1%	91.8%
*F* = 19.3; *p* < 0.0001		c	b	c	b	c	ab	b	a
Taste	**−**	22.2%	11.5%	28.1%	14.9%	26.5%	13.9%	17.2%	9.4%
	+	77.8%	88.5%	71.9%	85.1%	73.5%	86.1%	82.8%	90.6%
*F* = 14.7; *p* < 0.0001		de	ab	e	bc	e	c	cb	a
Mouthfeel	**−**	56.8%	35.1%	44.4%	32.2%	30.8%	16.0%	27.5%	20.2%
	+	43.2%	64.9%	55.6%	67.8%	69.2%	84.0%	72.5%	79.8%
*F* = 28.8; *p* < 0.0001		e	d	d	bc	bc	a	b	a

*Note:* Different letters indicate significant differences between countries and participants previous exposure. The + (coloured in green) and − (coloured in red) symbols indicate whether the perception of each sensory dimension was described as positive or negative.

Abbreviations: Co, Conceptual; Pe, Perceptual.

### 3.2 Is There a Cross‐Cultural Influence on the Overall Seaweed Sensory Space?

The comparative representation of the overall similarities, and differences, associated with the participants self‐assessments of seaweed sensory space can be found in Figure 1. The variability between participants (i.e., perceptual and conceptual for each country) is represented through projected points (Figure 1a), which represent the four sensory dimensions (color—C, flavor—F, basic tastes—T, and mouthfeel—M). Through these projected points, we can observe how these four sensory dimensions contribute to country separation based on their positioning in the given space.

**FIGURE 1:**
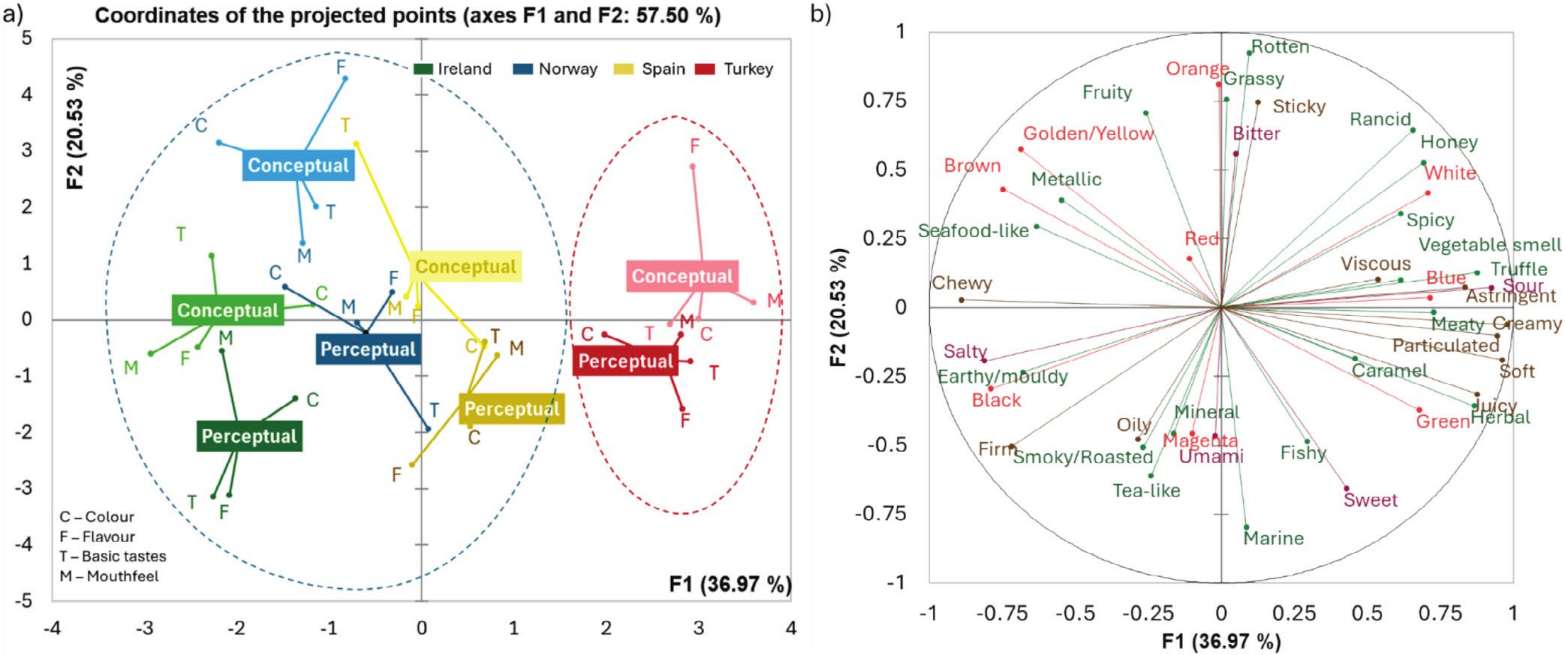
(a) Multiple Factor Analysis (MFA) showing coordinates of the projected points for each country and participants exposure (perceptual and conceptual). Perceptual and Conceptual labels of a same country are displayed with a similar color hue (Ireland—green, Norway—blue, Spain—yellow, and Turkey—red). Abbreviations in (a) indicate the separate data tables as follows: C (color), F (flavors), T (basic tastes) and M (mouthfeel). (b) Loading plots showing the distribution of individual sensory variables colored according to the different data tables.

From the results, it can be extracted that sensory associations are influenced by the participants country of origin. The cumulative explained variance (EV%) achieved between the first two dimensions is of 57.50% (F1: 36.97% and F2: 20.53%), and these differences between countries can be mainly observed along F1. The ovals correspond with the cluster formations obtained from Agglomerative Hierarchical Clustering on MFA dimensions, showing that. Turkish seaweed sensory space clustered on its own, whereas Ireland, Norway and Spain clustered together. The distribution of the observations alongside the sensory space is explained with the sensory attributes shown in the correlation map in Figure 1b. The direction of the variables (i.e., sensory attributes) indicates their direction, and contribution toward observation separation in Figure 1a.

The sensory attributes “sour” (right side of F1b) and “salty” (left side of F1b) are the main contributors to this separation along F1, being drivers of separation between countries. This translates to a higher rFoC of “sour”, and lower rFoC of “salty” by Turkish participants, compared to Spain, Norway, and especially Ireland. Conversely, if we analyze the separation along the vertical axis (F2), we can identify a pattern where separation conceptuals are on the positive side of the vertical axis and perceptuals are on the negative side (Figure 1a). The largest contributor to this separation is the term “sweet”, followed by “bitter”, “orange” (color), “rotten”, and “umami”. Whilst the attributes “sweet” and “umami” are associated with perceptuals, “bitter”, “orange” (color), and “rotten” are associated with conceptuals. It is possible that this differentiation reflects, at least in part, semantic or linguistic factors rather than a purely perceptual difference, as cross‐linguistic variation in the application of basic taste terms has been documented in prior literature (Osawa and Ellen 2014). The survey design, which captured positive/negative perception of each sensory dimension separately from the selection of individual associated attributes, may partially mitigate this risk, though it cannot be fully excluded.

A closer examination of Figure 1a, shows that F (i.e., flavor) dimensions contribute to a larger separation among observations. In the case of Ireland, Norway, and Spain, the projections associated to F in perceptuals and conceptuals pull toward the same axis direction. The opposite trend is observed for Turkey as F (conceptuals) is contributing to the separation toward the positive side of F2 whereas it pulls toward the negative side of F2. This could be a first indicator that larger differences associated to flavor could be identified between Turkish perceptual and conceptual participants. These individual sensory dimensions will be analyzed in detail in the following sections.

### 3.3 In‐Depth Sensory Insights From Analysis on Individual Attributes

A more in‐depth assessment was conducted to understand the role of individual sensory attributes within their corresponding sensory dimension (color, flavor, basic tastes, and mouthfeel). ANOVA results showed that there is a significant impact of country and participants' past exposure (i.e., perceptual and conceptual) on all sensory attributes except for two colors (white and magenta). These differences are displayed in Figure 2 and Figure 3.

**FIGURE 2:**
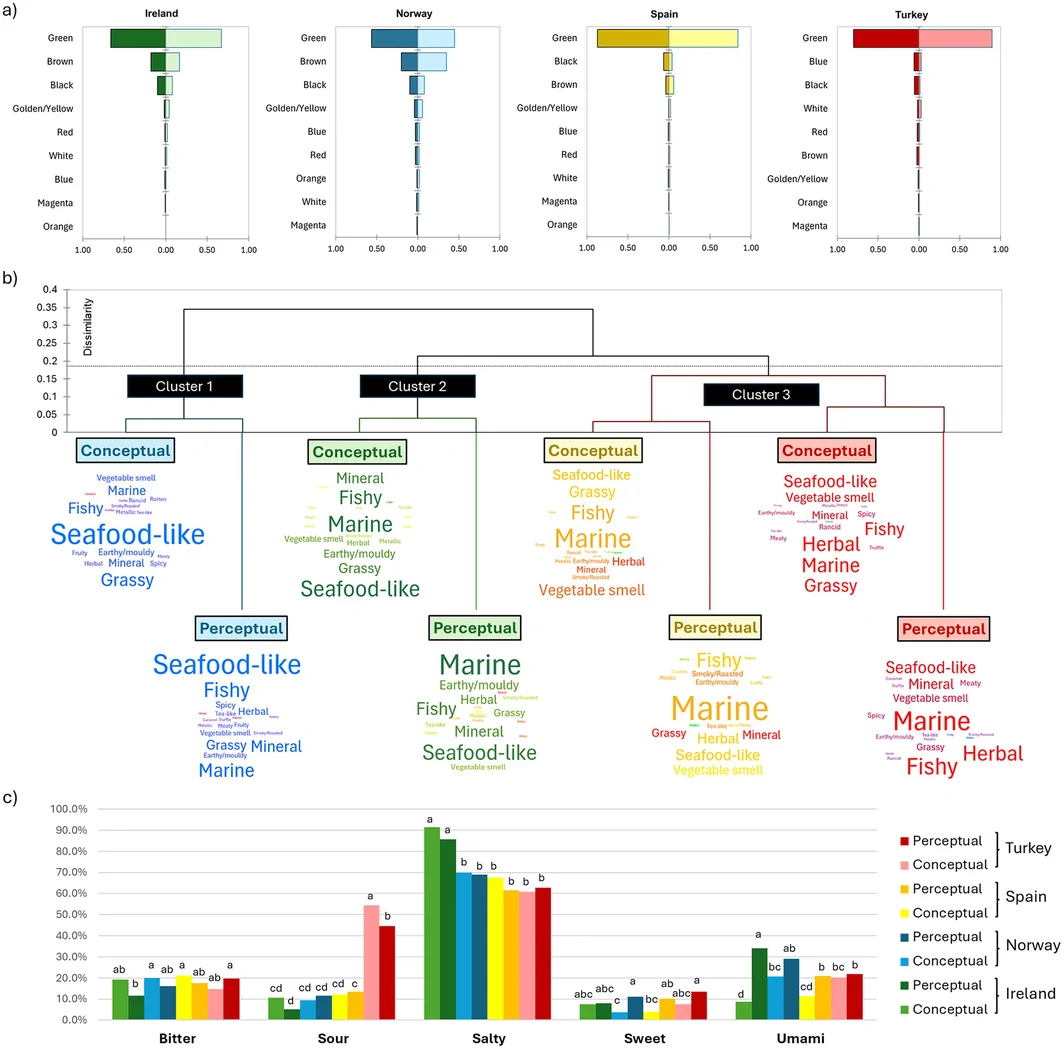
(a) Butterfly diagrams illustrating color associations according to the different markets and participants past experiences. (b) Agglomerative Hierarchical Clustering (AHC) on flavor average values to unveil overall similarities and differences for this sensory dimension. The sensory attributes for each market and participants experience are illustrated with wordclouds based on their relative Frequency of Citation (c) Bar plots illustrating differences between countries and exposure on how participants associated basic tastes with seaweed. Different letters indicate significant differences of mean values.

**FIGURE 3:**
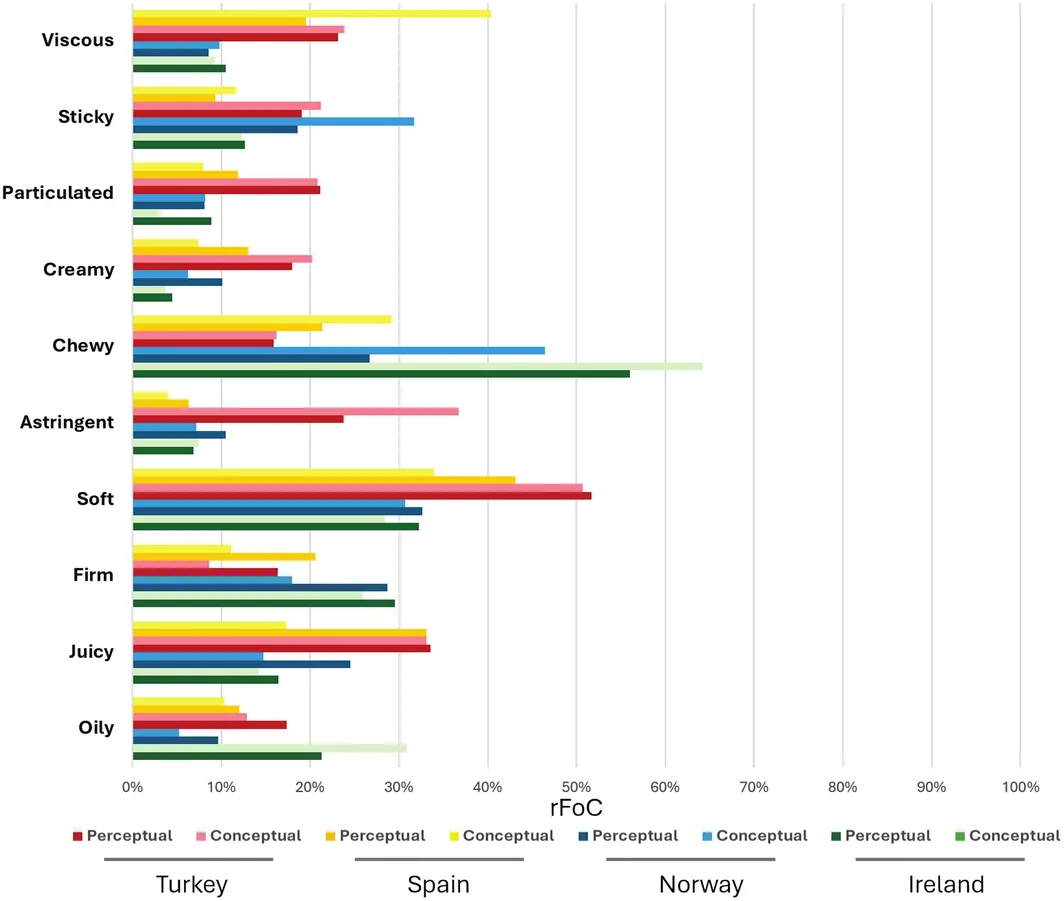
Bar plot showing the differences between countries and past experiences regarding participants' associations between texture sensory properties and seaweed captured using Check‐All‐That‐Apply. Bars are colored according to each market, with a different tonality of the same color to differentiate conceptuals (light) and perceptuals (dark). rFoC stands for relative Frequency of Citation.

#### 3.3.1 Color

A set of four butterfly diagrams are shown in Figure 2a. These charts compare the rFoC of each color between perceptual and conceptual answers for each country. Results showed that “green” was the color with the highest citation in perceptuals and conceptuals from all four countries. These percentages were above 80% in Spanish (87% perceptuals and 84% conceptuals) and Turkish participants (80% perceptuals and 90% conceptuals). A larger variability was observed in participants from Ireland and especially Norway. Participants from these two countries also selected “brown” (between 17%– 35%) and to a lower extent, “black” (5%–10%). A residual number of citations was found for all the remaining colors (≤ 6%) (Figure 2a). For the color “brown”, the proportion between perceptual and conceptuals was balanced in Ireland. However, a larger % of Norwegian participants who had never eaten seaweed (35%) considered “brown” as their first association compared to those participants who had eaten seaweed (20%). This diversity in color associations may be influenced by specific seaweed species integrated within food products, or by participants' visual familiarity with seaweed and their presence along the coastline. For example, the presence of *Saccharina latissima* and *Laminaria digitata* (brown seaweed) is common in the Atlantic Ocean but its presence in the Mediterranean is rare (Jaugeon et al. 2025; MarLIN—The Marine Life Information Network, n.d.).

It is important to bear in mind that the question about color limited the range of possible responses, encouraging participants to give the first answer that came to mind. Therefore, the fact the Irish and Norwegian participants selected other colors does not imply that they do not associate the color “green” with seaweed. A larger variability could also be an indicator of a potential larger seaweed diversity within those countries.

#### 3.3.2 Flavor

Based on the outcome from the MFA, flavor could be driving the difference between countries. To get a deeper understanding, ANOVA analysis was conducted. Additionally, AHC was performed on the raw data to investigate the similarities between countries based on just the flavor dimensions.

As displayed in Figure 2b, three clusters were obtained based on the selected flavors. Flavor associations seem to be highly influenced by the participants' country of origin as in all cases, “perceptuals” and “conceptuals” of each country cluster together. Cluster 1 groups Norwegian “perceptual” and “conceptual”, Cluster 2 groups Irish “perceptual” and “conceptual”. Lastly, Cluster 3 groups together the flavor dimension described by Spanish and Turkish participants.

ANOVA showed significant differences between countries and experiences for all sensory attributes. Based on the F values, the top‐five flavor attributes contributing to the differences between countries and experiences were “marine” (*F* = 74.16; *p* = < 0.0001), “herbal” (*F* = 50.97; *p* = < 0.0001), “seafood‐like” (*F* = 28.72; *p* = < 0.0001), “spicy” (*F* = 24.97; *p* = < 0.0001), and “earthy/moldy” (*F* = 24.52; *p* = < 0.0001). The wordclouds in Figure 2b help to visualize the contribution of these terms represented by their rFoC.

The term “marine” shows a larger rFoC in Spain and Turkey, as well as in Ireland, when compared to Norway. This attribute, however, is influenced by participants' past experiences. A significantly higher relative citation was associated with perceptuals from all countries, with 79.5% of Spanish perceptuals citing it as a flavor attribute associated with seaweed. In contrast, only 37.4% of Norwegians' perceptuals considered it applicable. In the case of “herbal”, this term mainly drives the differences between Turkey and the other three markets with > 50% of Turkish participants (both perceptual and conceptual) associating this term with seaweed. Besides Turkey, only Irish perceptuals showed a rFoC > 30% (30.6%) as opposed to the 17.9% of Irish conceptuals.

The term “seafood‐like” (Figure 2b) shows a larger association to seaweed in Norway compared to Turkey, and especially Spain. Within the answers from all Norwegian participants, conceptuals (66.0%) have a significantly higher rFoC than perceptuals (55.8%). A strong association of the term “seafood‐like” was also found for Irish participants, irrespective of their previous experience.

The case of “spicy” and “earthy/moldy” differed between countries and is driven by a very low citation of these terms by certain groups. Under 5% of Irish and Spanish participants associated it with seaweed (perceptuals and conceptuals), whereas a range between 12.4% and 16.9% of Norwegians and Turkish participants considered seaweeds to be “spicy.” Similarly, around 30% of Irish participants consider seaweed to be “earthy/moldy” (perceptuals—30.5%, conceptuals 29.6%), followed by Norwegian participants who had never eaten seaweed (17.9%).

#### 3.3.3 Basic Tastes

Differences between countries and past experiences were also found for basic tastes. As observed in Figure 2c, “salty” was the basic taste with the largest rFoC. This rFoC was significantly higher in Ireland (perceptuals—85.8%, and conceptuals—91.4%) compared to participants from the other three countries. For Norway, Spain, and Turkey, a range between 60%–70% participants of each group considered seaweed to be “salty.” It is therefore clear that European consumers have a strong association toward seaweed and the derived products being characterized by a high saltiness.

It is worth to highlight the differences found for the other tastes. A larger percentage of Turkish participants selected “sour” as a basic taste associated to seaweed (perceptual—44.6%, and conceptual—58.6%). Small differences were also found for “sweetness” and “bitterness” but with lower percentage. However, in the case of “umami”, perceptuals showed a larger rFoC than their corresponding conceptuals. These differences were statistically significant in Ireland and Spain; however, the trend is present in all countries. In the case of Ireland, 34.1% of Irish perceptuals checked the term “umami” as a sensory attribute associated to seaweed, in contrast to the 20.9% of Irish conceptuals.

#### 3.3.4 Texture Mouthfeel

Table 2 showed how texture was perceived as one of the most negative sensory dimensions associated to seaweed, notably among those participants who had never eaten seaweed before. This % was high for Ireland where a larger amount of conceptuals perceived it as a negative factor (56.8%). Looking at Figure 3, we can identify immediate differences between countries, and this was confirmed with Tukey's HSD post hoc test on ANOVA mean values (Table S2).

A large % of Irish participants consider seaweed as “chewy”, this was significantly higher in conceptuals (64.2%) than perceptuals (56%), followed by conceptual Norwegians (46.4%). In Spain, there was also a significantly higher association by conceptuals, whereas in Turkey there was no difference between these two groups. The terms “oily” and “firm” were found to be significantly larger in Ireland. On the other hand, a lower rFoC was associated with “viscous”, “sticky”, “astringent” nor “creamy”.

For Turkish participants, “soft” and “juicy” were the most frequent attributes. Likewise in Spain, a large % of participants considered seaweed as “soft” and “viscous”. The term “soft” showed a significantly lower rFoC among Irish and Norwegians. Figure 4 shows how a larger amount of Turkish conceptuals selected “astringent” as a sensory attribute. All these textural parameters would be linked to types of process applied to seaweed and type of food products. Therefore, exposure to specific type of products may be influencing those associations. However, it may also be understood differently because people develop different cultural associations.

**FIGURE 4:**
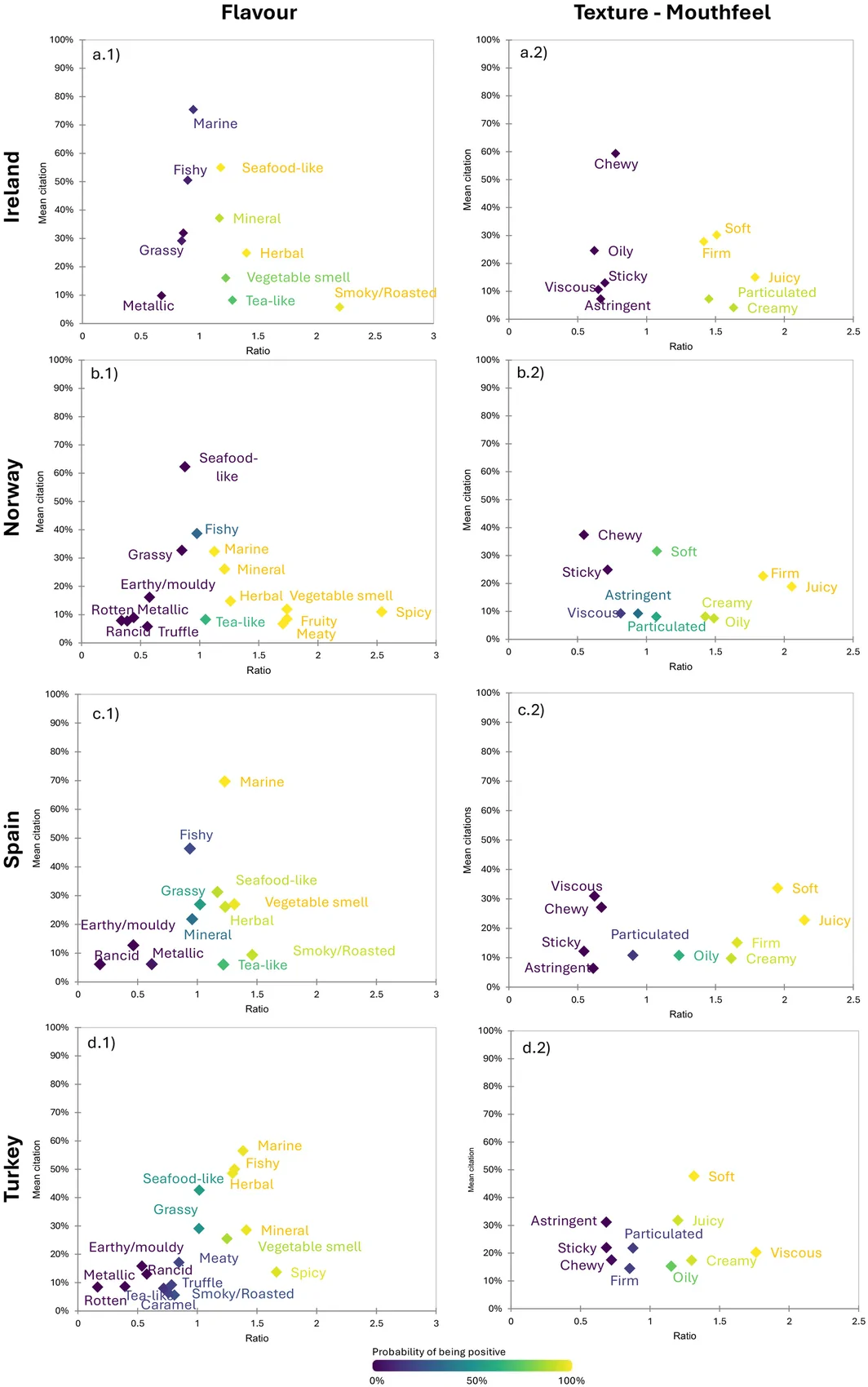
Sensory attribute polarity ratio for flavor and texture‐mouthfeel properties. Positive attributes are colored in green, negative in red, and in yellow polarizing sensory attributes.

### 3.4 Exploring Patterns on How Individual Sensory Properties Associate to Positive or Negative Perceptions

We have explored if some of the individual attributes, namely flavor and texture‐mouthfeel, are associated with positive or negative perception of their corresponding sensory dimensions shown in Table 2. Other parameters such as color and basic tastes were not explored. This was decided based on the fact that color was limited to the first association that came to mind, with a large association to green in all cases; and taste was limited to the five basic taste options. Additionally, there was no evaluation of self‐perceived appearance descriptors.

For flavor and texture‐mouthfeel, the citation counts of each individual attribute were compared between participants who perceived the corresponding sensory dimension (i.e., flavor and texture‐mouthfeel) as positive and those who perceived it as negative. The probability of each remaining attribute being positive was then estimated using a Bayesian Beta‐Binomial model. In Figure 4, colors represent the probability of the attribute being associated to a positive perception of the dimension using a continuous gradient scale from purple (i.e., low probability) to yellow (i.e., high probability).

Similarities are observed between countries. The term “metallic” was associated with a negative perception in all countries. In the case of Ireland, more sensory attributes with a lower rFoC were excluded. In Norway, “grassy”, “earthy/moldy”, “rotten”, “rancid”, and “truffle” were also associated with a negative perception (Figure 4b.1). In Turkey, other attributes such as “meaty”, “tea‐like”, or “smoky roasted” did not show a high probability of being perceived as positive. Additionally, in Turkey, these “negative” attributes tend to have a lower rFoC when looking at the vertical axis.

Looking at the positive, “vegetable‐smell” shows a high probability of being associated with a positive perception, especially in Norway and Spain. Similarly, in Ireland and Spain, “seafood‐like” shows a positive perception. However, this is an example of a “polarizing attribute” in Norway and Turkey. The attribute, despite having a medium to low probability of being associated with positive flavor perception, has a considerable rFoC (Norway—62% of participants, Turkey—43%). Therefore, in these two countries, if a product developer wants to have a seaweed‐based product with “seafood‐like” flavor, they should expect a large proportion of consumers to perceive it positively despite the low Bayesian probability. The case of “fishy” and “marine” are similar. The term “fishy” showed a high probability of being associated with a positive flavor dimension only in Turkey. For “marine”, it was in Norway, Spain, and Turkey but not in Ireland where, despite having a high rFoC between positive and negative groups (73% vs. 77%), the Bayesian probability indicated a weaker association with positive flavor perception (20%).

From a texture perspective, similar patterns are observed across countries. The terms “chewy” and “sticky” were associated with a negative perception of seaweed texture properties. In Ireland, “chewy” is polarizing, as it showed a high rFoC. In the case of “viscous” it was negative for Ireland, Norway, and Spain, but positive for Turkish participants.

The term “creamy” was associated with a positive texture‐mouthfeel dimension in all four countries whereas “soft” had a lower probability of being associated with a positive texture‐mouthfeel in Norway than in the other three (Figure 4b). The term “firm” had a low probability of being a positive attribute only in Turkey. Cross‐cultural differences between “soft” and “firm” were already shown in a study comparing Chinese and Danish populations (Cattaneo et al. 2020). In that study, most Chinese consumers (77%) were described as “soft processing likers” with a higher preference for soft foods, whereas most Danish were “firm processing likers” (73%).

Other cultural differences were found for “oily”. Whilst negatively associated in Ireland, it showed a higher citation among those participants who considered texture as positive in Norway, Spain and Turkey. In any cross‐cultural study, especially in countries with different languages, there is always a risk of misinterpretation or different semantic association to individual terms. Texture lexicon is challenging, and it has been shown that these could be linked to language (Kumar and Chambers 2019) and cultural differences on how participants understand mouthfeel properties (Jeong and Lee 2021; Rizo and Tárrega 2023).

## 4 Conclusion

The current work explored the sensory space associated with seaweed among consumers willing to try seaweed products across four European countries. Results confirmed that country of origin and previous exposure to seaweed products significantly influenced sensory associations across all five organoleptic dimensions evaluated and individual sensory attributes. Consumer segmentation could have been approached following different criteria such as eating behaviors, food liking, or direct familiarity with seaweed material.

These findings offer valuable insights for product developers regarding which organoleptic properties consumers associate with seaweed. In our results, “green” was the dominant color association in all four countries, though a larger diversity of color citations was observed in Ireland and Norway, likely reflecting the presence of brown seaweed species along Atlantic coastlines. For flavor, country‐driven clustering was evident. “Marine” and “seafood‐like” were among the most cited flavor attributes, with perceptuals showing consistently a relatively higher citation frequency than conceptuals. “Salty” was the dominant basic taste across all markets, strengthening the known potential of seaweed as an alternative salt replacer. For texture‐mouthfeel, “chewy” was predominantly associated with a negative perception, whereas “creamy” and “soft” showed positive associations across markets.

The polarity analysis further highlighted how certain sensory attributes have a consistent negative valence as for example “metallic”, “chewy” and “sticky”. These attributes could act as barriers to acceptance, that could be mitigated through processing choices (e.g., particle size reduction, flavor masking, or texture modification), whereas other attributes with positive valence such as “marine”, “creamy” and “seafood‐like” may serve as drivers of liking and could be emphasized in product formulation and messaging.

### 4.1 Limitations and Future Perspectives

The current consumer segmentation focuses on self‐reported previous consumption. However, there might be participants who self‐reported themselves as not having consumed seaweed products (“conceptuals”) but have consumed derived seaweed ingredients. Therefore, the current study could be approached from different angles, with a further consumer segmentation based on exposure to raw material, seaweed hydrocolloids or ingredients (e.g., carrageenan, alginates), and seaweed food products. Additionally, participants level familiarity with different seaweed species could play a role (number and diversity of seaweed species). This information was not captured, and it could be integrated in future studies through images or relevant questions.

Nonetheless, these findings provide a useful foundation for product developers aiming to navigate the seaweed sensory space across European markets, where cultural exposure and individual experience with seaweed products remain key factors shaping consumer perception. Product developers can take on the most frequent organoleptic associations and their potential positive perception to be tested in future central‐location‐test (CLT) studies with real prototypes.

## Author Contributions

**Gonzalo Garrido‐Bañuelos:** conceptualization, investigation, writing – original draft, funding acquisition, methodology, visualization, writing – review and editing, formal analysis, data curation. **Guro Møen Tveit:** conceptualization, writing – original draft, writing – review and editing, funding acquisition. **Hanne Dalsvåg:** conceptualization, writing – original draft, writing – review and editing. **Solveig Uglem:** conceptualization, writing – original draft, writing – review and editing. **Marta Martínez‐Sanz:** conceptualization, funding acquisition, writing – original draft, writing – review and editing. **Patricia Lopez‐Sanchez:** conceptualization, funding acquisition, writing – original draft, writing – review and editing.

## Funding

This work is part of the SEAFOODTURE project (Integral Valorisation of Seaweed Biomass for the Development of Sustainable, High Nutritional Quality Food Products), financially supported by the Sustainable Blue Economy Partnership (SBEP) and the different local entities: Marine Institute (MI) in Ireland (PBA/SBEP/24/03), Research Council of Norway (funded under project number 351740), and by Agencia Estatal de Investigación (PCI2024‐153420, PCI2024‐153421).

## Conflicts of Interest

The authors declare no conflicts of interest.

## Supporting information


**Table S1:** Summary of survey demographic data with total numbers and representative percentage to each country.
**Table S2:** Percentage distribution of responses across groups (country * exposure) based on two‐way ANOVA. Different letters indicate significant differences according to Tukey's HSD test.

## Data Availability

The data that support the findings of this study are available from the corresponding author upon reasonable request.
