# Supplementary material for: Toward a Deeper Understanding of the Seaweed Sensory Space Through Consumer‐Evoked Associations Across Ireland, Norway, Spain, and Turkey
Source: Food Sci Nutr. 2026 Oct 26;14(11):e72508. doi: 10.1002/fsn3.72508 (PMC13710795; doi:10.1002/fsn3.72508)
Supplement: Supplementary file 1 [file FSN3-14-e72508-s001.docx]

Supplementary Table 1. Summary of survey demographic data with total numbers and representative percentage to each country.

|  | **Ireland** | **Norway** | **Spain** | **Turkey** |
| --- | --- | --- | --- | --- |
| Female | 410 (57.18%) | 346 (45.35%) | 542 (50.42%) | 433 (49.57%) |
| Male | 306 (42.68%) | 415 (54.39%) | 533 (49.58%) | 504 (53.79%) |
| Non-binary | 1 (0.14%) | 2 (0.26%) | (0.00%) | (0.00%) |
| 18 - 24 | 36 (5.02%) | 133 (17.43%) | 106 (9.86%) | 128 (13.66%) |
| 25 - 34 | 142 (19.80%) | 194 (25.43%) | 165 (15.35%) | 290 (30.95%) |
| 35 - 44 | 243 (33.89%) | 164 (21.49%) | 289 (26.88%) | 253 (27.00%) |
| 45 - 54 | 182 (25.38%) | 152 (19.92%) | 312 (29.03%) | 174 (18.57%) |
| 55 - 64 | 85 (11.85%) | 86 (11.27%) | 176 (16.37%) | 75 (8.00%) |
| 65+ | 29 (4.04%) | 34 (4.46%) | 27 (2.51%) | 17 (1.81%) |
| **Total** | **717** | **763** | **1075** | **937** |

Supplementary Table 2. Percentage distribution of responses across groups (country * exposure) based on two-way ANOVA. Different letters indicate significant differences according to Tukey´s HSD test.

| **Country** | **Exposure** | **Oily** | **Juicy** | **Firm** | **Soft** | **Astringent** | **Chewy** | **Creamy** | **Particulated** | **Sticky** | **Viscous** |
| --- | --- | --- | --- | --- | --- | --- | --- | --- | --- | --- | --- |
| Ireland | Pe. | 21% b | 16% c | 30% a | 32% c | 7% cd | 56% b | 5% d | 9% b | 13% c | 10% c |
| Ireland | Co. | 31% a | 14% c | 26% ab | 28% c | 7% cd | 64% a | 4% d | 3% c | 12% c | 9% c |
| Norway | Pe. | 10% de | 25% b | 29% a | 33% c | 11% c | 27% d | 10% bc | 8% bc | 19% b | 9% c |
| Norway | Co. | 5% e | 15% c | 18% c | 31% c | 7% cd | 46% c | 6% cd | 8% bc | 32% a | 10% c |
| Spain | Pe. | 12% d | 33% a | 21% bc | 43% b | 6% d | 21% e | 13% b | 12% b | 9% c | 19% b |
| Spain | Co. | 10% de | 17% c | 11% d | 34% c | 4% d | 29% d | 7% cd | 8% bc | 12% c | 40% a |
| Turkey | Pe. | 17% c | 34% a | 16% c | 52% a | 24% b | 16% f | 18% a | 21% a | 19% b | 23% b |
| Turkey | Co. | 13% cd | 33% a | 9% d | 51% a | 37% a | 16% ef | 20% a | 21% a | 21% b | 24% b |
